# Editorial: Understanding and Exploiting Host-Commensal Interactions to Combat Pathogens

**DOI:** 10.3389/fimmu.2019.02645

**Published:** 2019-11-12

**Authors:** Sudhanshu Shekhar, Fernanda Cristina Petersen, Xi Yang

**Affiliations:** ^1^Faculty of Dentistry, Institute of Oral Biology, University of Oslo, Oslo, Norway; ^2^Department of Immunology, Max Rady College of Medicine, University of Manitoba, Winnipeg, MB, Canada

**Keywords:** commensal, host, immunity, pathogen, vaccine, therapeutics

The human body harbors an astonishing number of diverse commensal microbes, including bacteria, fungi, and viruses, providing a suitable milieu for microbial growth and multiplication (1, 2). These microbes rarely cause disease and communicate with the host in a mode that is advantageous to both host and microbes (3, 4). In recent years, phylogenetic, metagenomic, and functional studies have been conducted to better understand the complexities of the microbial genome and the effect of the microbiota on the host's immunophysiology. Accumulating evidence has shown that gut commensals regulate the ontogeny and function of the immune system, and contribute to shaping the outcome of immune responses (5). It has also become clear that these commensals have the ability to influence the immune responses at extraintestinal tissues/organs, underscoring their profound impact on local as well as systemic immunity (6). However, alterations in composition, diversity, and metabolic activities of commensal microbes can lead to dysbiosis, which may have detrimental consequences, such as autoimmunity, allergy, asthma, inflammatory bowel disease, cancer, and infection (7). Commensals also hold the potential to cause disease depending on multiple microbial and host factors (8). A much deeper understanding of how commensals communicate with the host is crucial for developing new strategies to prevent and treat diseases.

In this Research Topic, a series of 16 articles, encompassing review, original research, and general commentary articles, provide crucial information on how the interplay between host and commensals takes place and how this could be exploited for designing novel prophylactics/therapeutics against a wide spectrum of disorders, including infectious diseases. In an original research article, Yang et al. report the identification of genetic factors that are involved in the lysis of human neutrophils by *Staphylococcus aureus*, underscoring the mechanism by which this commensal bacterium evades the neutrophilic immune barrier during infection. Likewise, Dai et al. demonstrate that *S. aureus* employs the vancomycin resistance-associated sensor/regulator (VraSR) to increase its survival within macrophages, thereby modulating the process of host-cell autophagy. Overall, these data throw light on important virulence factors used by *S. aureus* to escape innate immunity, highlighting why the innate immune response is incapable of eradicating *S. aureus*. Gao et al. provide evidence that underscores the impact of hydrogen peroxide (H_2_O_2_) produced by *Streptococcus pneumoniae* on the host's immune responses against pneumococcal lung infection. *S. pneumoniae*-secreted H_2_O_2_ causes damage and leakage of mitochondrial DNA into the cytoplasm, which not only mediates mitochondrial oxidative stress but also promotes IFN-I cascades in lung cells (Gao et al.). Furthermore, Tian et al. show that gut microbiome-derived propionate levels are inversely proportional to the lung inflammation, but not to bacterial immunity, using mouse models of ischemia reperfusion injury and *S. aureus* pneumonia. These studies indicate that microbial products, such as H_2_O_2_ and propionates, play a significant role in the outcome of host immunity.

Luo et al. describes the inhibitory effect of human cathelicidin antimicrobial peptide LL37 on *Aspergillus fumigatus* infection in mice by directly binding to the fungal mycelia, which follows reduced pulmonary inflammation characterized by decreased histopathological changes and proinflammatory cytokine levels. Woo et al. show a novel mechanism by which the commensal microbiota epigenetically regulate intestinal epithelial cells to downregulate expression of the cell surface glycoprotein C-type lectin 2e (Clec2e), which reduces the efficiency of epithelial cell interaction with the murine enteric pathogen *Citrobacter rodentium*. On the other hand, host immunity can also regulate the composition of the mucosal-associated microbiota. Xiao et al. demonstrate that Toll-like receptor 4 (TLR4), which is a membrane-bound protein expressed on immune cells that identifies microbe-associated molecular patterns, may play a role in the regulation of the distribution and structure of the intestinal mucosal-associated microbiota by vitamin A. Thus, there exists a bidirectional communication between the microbiota and host, which may contribute to maintaining homeostasis in the gut.

The review and commentary articles presented in this Research Topic focus on a variety of interesting areas that include commensal-immune cell interaction and its implications for therapy and prophylaxis of diseases. Humbert et al. review the current literature on the pathophysiology of chronic mucocutaneous candidiasis, which is caused by the fungus *Candida albicans*, in autoimmune polyendocrine syndrome type 1, whereas Iacob and Iacob focus on the importance of the relationship between the intestinal barrier and microbiota, and how dysbiosis can alter this relationship to make the host prone to pathogens. Additionally, it is becoming clearer that the gut microbiota modulates the innate and adaptive immune responses to impact the disease outcome. Cheng et al. shed light on the major mechanisms by which the commensal microbiota boosts the host's innate immunity against infectious agents. Pandiyan et al. review recent evidence on how the gut microbiota influences the function of adaptive immune cells, such as regulatory T cells (Tregs) and Th17 cells, and how the microbiota can be targeted to promote mucosal immunity and ameliorate pathology. Natural killer T (NKT) cells constitute an innate and unconventional population of T cells that perform protective as well as detrimental roles in diverse disease models. A general commentary by Jia focuses on the crucial role played by NKT cells in liver cancer, which is regulated by the gut microbiota-mediated bile acid metabolism.

An interesting review by Forgie et al. discusses the host-microbiota interactions from a dietary point of view. This provides knowledge on how dietary components, such as carbohydrates and proteins, can modulate the host-microbiota interactions to promote resistance against pathogens (Forgie et al.) Studies on the therapeutic applications of the host-microbiota dialogue are crucial for ensuring further translation of the acquired knowledge into health benefits. Li et al. provides a comprehensive review on the beneficial and harmful role of the commensal microbiota in dealing with viral infections and the effect of these infections on the microbiota homeostasis. Khan et al. evaluate emerging data on the contribution of commensal bacteria to host defense against respiratory pathogens and the mechanisms whereby bacteria induce protective immunity. They also discuss how commensal bacteria can be exploited to treat and prevent respiratory infections (Khan et al.). In line with this, Baker and Edlund discuss the therapeutic potential of the oral microbiome in developing strategies to exploit the protective effects of the oral microflora in order to prevent dental caries.

Cumulatively, this Research Topic provides significant knowledge on the mechanisms underlying host and commensal microbe interactions, and the profound impact that these microbes exert on the host's health and disease. This has clinical implications for the prevention and treatment of diseases. The targeting of commensals is gaining momentum as an effective strategy to combat various diseases, including infectious diseases. Successful treatment of severe intestinal infections, caused by antibiotic-resistant bacterial pathogens, using fecal microbiota transplantation, offers an excellent example of how commensal microbes can be used for disease therapy. It is, however, notable that most data on this topic stem from mouse studies. Although mouse experiments remain critical to understand the contribution of the commensal microbiota to health and disease, they pose a concern for the scientific community because of their poor recapitulation of human conditions. For example, the murine and human intestinal microbiota exhibit significant differences in abundance and gene identity, and the murine microbiota composition depends on multiple factors such as rearing facilities and genetic background (9). It is important to consider these facts while translating the knowledge acquired from mouse to human.

Since several commensals/probiotics have been shown to be safe in animal models and humans, it would be worth examining the long-term consequences of their use for the host's well-being *in toto*. With technological advances in this field, approaches that harness the beneficial effects of commensals to prevent diseases and promote health will continue to grow in number. In-depth studies are also needed to focus on the pathogenic potential of commensals/probiotics in individuals under immunosuppression due to malnutrition, chemotherapy, or viral infections. This is crucial because immunocompromised individuals possess an altered microbiota, along with an impaired immune system, which make them highly susceptible to opportunistic infections and cancer.

Finally, we are extremely grateful to all the authors for their significant contribution to this Research Topic as well as to the reviewers for taking the time to review the submitted manuscripts.

## Author Contributions

All authors wrote the editorial manuscript, and approved it for publication.

### Conflict of Interest

The authors declare that the research was conducted in the absence of any commercial or financial relationships that could be construed as a potential conflict of interest.

## References

[1] Gevers D Knight R Petrosino JF Huang K McGuire AL Birren BW. The Human Microbiome Project: a community resource for the healthy human microbiome. PLoS Biol. (2012) 10:e1001377. 10.1371/journal.pbio.1001377 22904687 PMC3419203

[2] Turnbaugh PJ Ley RE Hamady M Fraser-Liggett CM Knight R Gordon JI. The human microbiome project. Nature. (2007) 449:804–10. 10.1038/nature06244 17943116 PMC3709439

[3] Abt MC Pamer EG. Commensal bacteria mediated defenses against pathogens. Curr Opin Immunol. (2014) 29:16–22. 10.1016/j.coi.2014.03.003 24727150 PMC4132187

[4] Kamada N Seo SU Chen GY Nunez G. Role of the gut microbiota in immunity and inflammatory disease. Nat Rev Immunol. (2013) 13:321–35. 10.1038/nri3430 23618829

[5] Cerf-Bensussan N Gaboriau-Routhiau V The immune system and the gut microbiota: friends or foes? Nat Rev Immunol. (2010) 10:735–44. 10.1038/nri2850 20865020

[6] Samuelson DR Welsh DA Shellito JE. Regulation of lung immunity and host defense by the intestinal microbiota. Front Microbiol. (2015) 6:1085. 10.3389/fmicb.2015.01085 26500629 PMC4595839

[7] Carding S Verbeke K Vipond DT Corfe BM Owen LJ. Dysbiosis of the gut microbiota in disease. Microb Ecol Health Dis. (2015) 26:26191. 10.3402/mehd.v26.26191 25651997 PMC4315779

[8] Henriques-Normark B Normark S. Commensal pathogens, with a focus on *Streptococcus pneumoniae*, and interactions with the human host. Exp Cell Res. (2010) 316:1408–14. 10.1016/j.yexcr.2010.03.003 20227406

[9] Hugenholtz F de Vos WM. Mouse models for human intestinal microbiota research: a critical evaluation. Cell Mol Life Sci. (2018) 75:149–60. 10.1007/s00018-017-2693-8 29124307 PMC5752736

